# Effect of Sensor Size, Number and Position under the Foot to Measure the Center of Pressure (CoP) Displacement and Total Center of Pressure (CoPT) Using an Anatomical Foot Model

**DOI:** 10.3390/s23104848

**Published:** 2023-05-17

**Authors:** Hussein Abou Ghaida, Luiz Poffo, Ronan Le Page, Jean-Marc Goujon

**Affiliations:** Univ Rennes, CNRS, Institut FOTON-UMR 6082, 6 rue de Kerampont CS 80518, F-22305 Lannion, France

**Keywords:** pressure measurement, pressure sensors, sensor systems and applications, position measurement, center of pressure, plantar pressure measurement

## Abstract

Ambulatory instrumented insoles are widely used in real-time monitoring of the plantar pressure in order to calculate balance indicators such as Center of Pressure (CoP) or Pressure Maps. Such insoles include many pressure sensors; the required number and surface area of the sensors used are usually determined experimentally. Additionally, they follow the common plantar pressure zones, and the quality of measurement is usually strongly related to the number of sensors. In this paper, we experimentally investigate the robustness of an anatomical foot model, combined with a specific learning algorithm, to measure the static displacement of the center of pressure (CoP) and the center of total pressure (CoPT), as a function of the number, size, and position of sensors. Application of our algorithm to the pressure maps of nine healthy subjects shows that only three sensors per foot, with an area of about 1.5 × 1.5 cm^2^, are needed to give a good approximation of the CoP during quiet standing when placed on the main pressure areas.

## 1. Introduction

The measurement of plantar pressures and Center of Pressure (CoP) displacement has become an essential clinical diagnostic tool performed by podiatrists in order to detect improper pressure distribution [1,2] and to diagnose balance disorders [3]. In addition, studies may focus on athletes [4,5,6], diabetic patients [7,8], and the elderly [9].

The two most popular instrumented insole systems used by podiatrists are based on instrumented insoles with hundreds of sensors, such as the F-Scan Mobile® system [from Tekscan, Inc. Boston, MA. 02127-1309, USA] with 954 sensors or the Pedar® system [from Novel, Gmbh novel GmbH, Ismaninger Str. 51, D-81675 Munich] with 256 sensors. These systems monitor plantar pressure in both stance and ambulation [10]. However, plantar pressure monitoring is limited to a few hours due to the power consumption relative to battery storage capabilities. Moreover, the cost of these systems is relatively high. 

The best way to improve the system and reduce cost and power consumption is to reduce the number of sensors in the insole. Numerous aspects of insole monitoring systems have been discussed, including the number of sensors [11]. Simplified instrumented insoles with three to ten [12] or sixteen [13] pressure sensors per foot have been developed. Sensors are placed mainly under the heel, midfoot, and forefoot for clinical gait analysis [14] or to detect gait phases [15]. From these studies, it can be noted that the position and size of the sensors are essential criteria for measuring plantar pressure.

A study by Farnoosh et al. [16] investigated the effect of pressure sensor position, number, and size on plantar pressure measurement during walking using Compressive Sensing techniques. They found that reconstruction errors increase unacceptably for sets of less than four sensors, with acceptable robustness achieved from ten sensors per foot. In a more recent study, learning strategies were investigated in systems utilizing nine sensors per foot [17].

The aim of this study is to experimentally validate our method [12], which utilizes an anatomical model of the foot coupled with a learning algorithm capable of generating plantar pressure maps in a standing position with three discrete pressure sensors per foot.

In this paper, we demonstrate the robustness of our algorithm in terms of static CoP and the center of total pressure (CoPT) measurements, as compared to the results obtained from plantar pressure maps (F-Scan), with respect to the number, size, and position of sensors. The CoPT is a powerful indicator of balance maintenance, and physicians may consider various statistical parameters, such as the total area, speed, and symmetry of the CoP temporal line draw, to assess balance evolution or impairment. Accuracy will be assessed by the Root Mean Square (RMS) error found on the CoP displacement under each foot, or the CoPT of the body. The advantage of anatomical models combined with machine learning is that the physician can easily understand the parameter and values. 

## 2. Materials and Methods

### 2.1. Anatomical Foot Model

**Figure 1:**
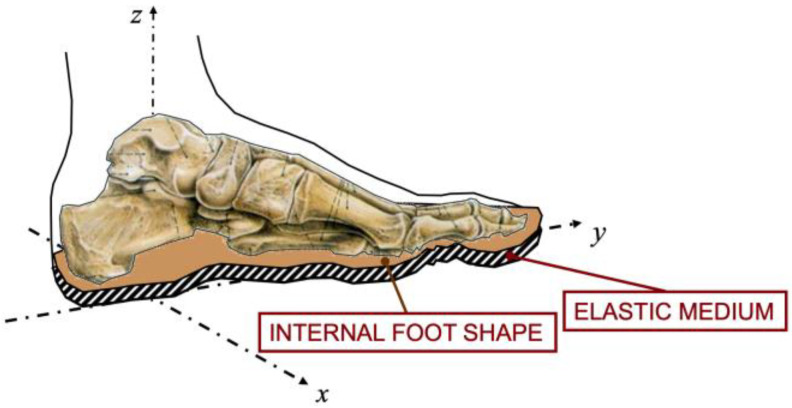
Foot model: foot seat, internal foot shape, and elastic medium.

The model establishes a relationship between posture and plantar pressure through mechanical foot characteristics. It is detailed in [12], and in summary, it takes into account three elements, shown in Figure 1:

- FS—the foot seat is the location of the foot in space given by the position of the ankle and the rotations around it;
- IS—the internal foot shape is a surface profile that describes the non-compressive material. The internal foot shape consists of the foot skeleton covered by other rigid media, such as the ligaments and the muscles;
- EM—the elastic medium is the soft tissue that covers the internal foot shape. The thickness and the elasticity of the soft tissue depend on the underfoot location. Compression of the tissue induces pressure.

These parameters are foot dependent. The force applied to compress the foot seat can be calculated by:

(1) $$F=-stiff\cdot \ln\left(1-\frac{Z_{C}}{thick}\right)$$

where *Z_C_* (mm) is the compression of EM, *thick* (mm) is the thickness, *stiff* (N/cm^2^) is the stiffness, and *F* (N/cm^2^) is the pressure.

Since the foot seat is defined by the equation of a plane, only three sensors per foot are sufficient to measure the plantar pressure map using this model.

### 2.2. Material and Protocol

#### 2.2.1. Plantar Pressure Reference Measurement

We asked nine healthy subjects, eight males and one female, aged 40 ± 15 years, weighing 70 ± 30 kg, and measuring 175 ± 10 cm, to participate. The plantar pressure maps under each foot are recorded, at a frequency of 50 frames/second, using the popular F-Scan Mobile® system. During about 30 s, the posture has to vary during recording to build the database and extract individual foot model parameters. This variation is natural during walking, but when standing, the subjects are asked to exaggerate their postures in mediolateral and anteroposterior directions. The variation is to ensure enough displacement amplitude to cover the entire foot surface and extract the key parameters of the anatomical foot model (foot shape, elastic medium stiffness and thickness) and posture (foot seat, total force).

#### 2.2.2. Sub-Matrix Sensors

To limit errors due to the experimental measurement and the electronic assembly, sensor pressure values are extracted from experimental plantar pressure maps acquired by the F-Scan Mobile® system, and a sub-matrix of this map will be considered as a sensor response. Changing the architecture is as simple as selecting different locations and sizes of sub-matrix sensors. This ensures that recorded data will remain identical and allows rigorous comparison between architectures. 

Two sizes of square sensors are tested: a small sensor with an area of 1.5 × 1.5 cm^2^ corresponding to 9 F-Scan pixels, and a large sensor with an area of 2.5 × 2.5 cm^2^ corresponding to 25 pixels. The pressure measured by each sensor is given by the sum of the F-Scan pixels, which are covered by the sub-matrix sensor.

We tested different numbers of sensors under each foot, ranging from 2 to 6. The sensor locations correspond roughly to the high-pressure support zones under the foot. These locations were derived directly from the F-Scan pressure maps.

A sensor is fixed under the heel, and the positions of the other sensors can vary under the forefoot and the foot arch. The sensors under the forefoot are substantially fixed at the first, second, third, and fifth metatarsal head. Table 1 presents the anatomical sensors’ position under the foot with respect to the chosen number of sensors.

### 2.3. Coordinate System

**Figure 2:**
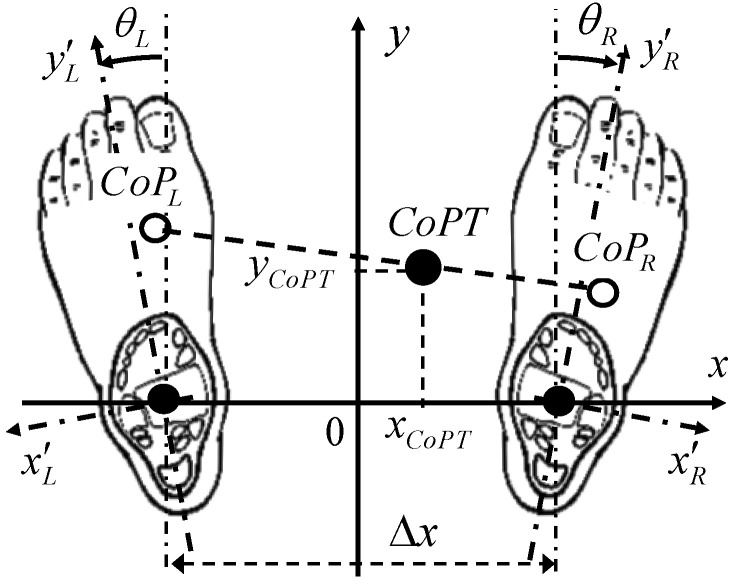
Schematic of the foot reference position used for Total Center of Pressure calculation. The x direction corresponds to the mediolateral movements, and the y direction corresponds to the anteroposterior movements.

In the quiet standing posture, the coordinate origin on the ground can be defined as the center distance between the ankles. The x direction, oriented from the left ankle to the right one, corresponds to the mediolateral movements. The distance between the ankles is Δx. The y direction is perpendicular and corresponds to the anteroposterior movements, as shown in Figure 2.

For each foot (L for left and R for right foot), the ankle is set as the origin of the internal coordinates. The directions $y_{L,R}^{\prime }$ are given by the ankle and the second metatarsus of each foot. 

The posture, depending on whether the feet are turned out or turned in, is defined by the angle $\theta_{L,R}$ made by the direction $y_{L,R}^{\prime }$ of each left and right foot with regard to the main (*y*) direction: $\theta_{L,R}$ > 0 for turned out and $\theta_{L,R}$ < 0 for turned in.

#### 2.3.1. Foot Center of Pressure from F-Scan Maps

For each foot, the available recorded data is the force $f_{i}^{k}$ applied to each pixel *k* = 1…*N* with coordinates $\left(x_{k}^{\prime },y_{k}^{\prime }\right)$ at each time step *i* = 1…*M*. Using the F-Scan system, the number of time steps is *M* ≈ 1200 for a 1 min recording and *N* = 950 pixels per foot.

At each time step, *i* = 1…*M*, we calculate the total force $F_{L,R}$ and the components of the moments $\overset{˘}{M}x_{L,R}^{\prime }$ and $\overset{˘}{M}y_{L,R}^{\prime }$ with respect to the ankle along the internal coordinates of the foot *x*′ and *y*′ by using linear forms:

(2) $$F_{i}=\sum_{k=1}^{N}f_{i}^{k}$$

(3) $$\overset{˘}{M}x_{i}^{\prime }=\sum_{k=1}^{N}x_{k}^{\prime }\cdot f_{i}^{k}\;\mathrm{and}\;\overset{˘}{M}y_{i}^{\prime }=\sum_{k=1}^{N}y_{k}^{\prime }\cdot f_{i}^{k},$$

#### 2.3.2. Foot Center of Pressure from Sparse Sensors

The number of sensors per foot is S. For each equilibrium time step *i*, the signal of the sensor *j* is $fc_{i}^{j}$. The main model hypothesis assumes linear forms to describe the moments and the total force from the sensors [18].

(4) $${\tilde{F}}_{i}=\sum_{j=1}^{S}Cf_{j}\cdot fc_{i}^{j}$$

(5) $$\tilde{\overset{˘}{M}}x_{i}=\sum_{j=1}^{S}Cmx_{j}\cdot fc_{i}^{j}\;,$$

(6) $$\tilde{\overset{˘}{M}}y_{i}=\sum_{j=1}^{S}Cmy_{j}\cdot fc_{i}^{j},$$

where *Cf_j_*, *Cmx_j_* and *Cmy_j_* are 3 sets of unknown constant coefficients to be determined for each of the *S* sensors per foot.

#### 2.3.3. Total Center of Pressure Calculation

The Total Center of Pressure (CoPT) is derived from the foot plantar pressure distribution. Therefore, the CoPT is a function of the foot position on the ground.

The moment components $\overset{˘}{M}x_{L,R}^{\prime }$, $\overset{˘}{M}y_{L,R}^{\prime }$ and the total force *F_L,R_* of each foot are determined from the F-Scan system, allowing the calculation of the CoPT of the body. The *x_CoPT_* and *y_CoPT_* coordinates of the CoPT derive directly from the two following relationships:

(7) $$x_{CoPT}=\frac{\overset{˘}{M}x_{L}^{\prime }\cdot \cos\theta_{L}+\overset{˘}{M}x_{R}^{\prime }\cdot \cos\theta_{R}-\overset{˘}{M}y_{L}^{\prime }\cdot \sin\theta_{L}+\overset{˘}{M}y_{R}^{\prime }\cdot \sin\theta_{R}}{F_{R}+F_{L}}+p$$

$$p=\frac{∆x}{2}\cdot \left(\frac{F_{R}-F_{L}}{F_{R}+F_{L}}\right)$$

(8) $$y_{CoPT}=\frac{\overset{˘}{M}y_{L}^{\prime }\cdot \cos\theta_{L}+\overset{˘}{M}y_{R}^{\prime }\cdot \cos\theta_{R}+\overset{˘}{M}x_{L}^{\prime }\cdot \sin\theta_{L}-\overset{˘}{M}x_{R}^{\prime }\cdot \sin\theta_{R}}{F_{R}+F_{L}}$$

In the standing posture, the feet position can be different from patient to patient. However, in practice, the foot gap Δx is usually about 20 to 25 cm. This distance is approximately equal to the width of the hips. The insoles were spaced 22 cm apart, which corresponds approximately to the natural position of the feet. During good posture, the feet are positioned so that they are slightly turned outwards at an angle of less than 20°.

## 3. Results

**Figure 3:**
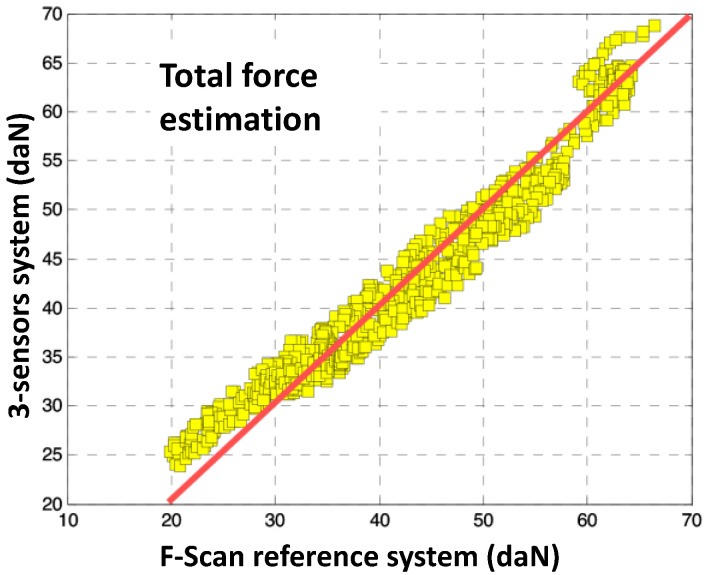
Comparison of the total force for 3 small sensors system using the anatomical model learning method with an F-Scan reference system.

First, we present in Figure 3 a comparison between the Total Force estimate measured by the F-Scan, and that calculated by the anatomical model using a set of three sensors.

### 3.1. Center of Pressure Measurement

**Figure 4:**
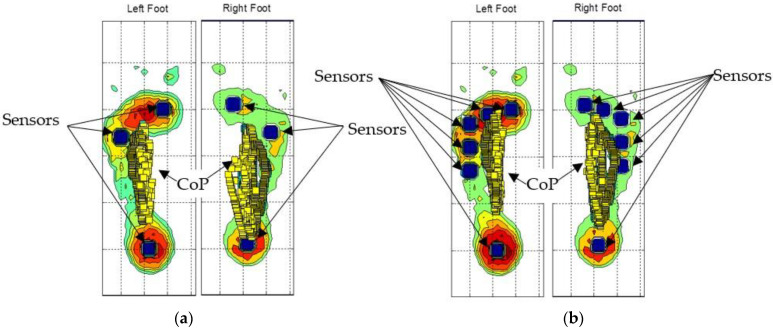
(**a**) Three sensors under each foot (blue squares); (**b**) six sensors under each foot. The CoPs are represented by yellow squares.

The sub-matrix sensors are represented by blue squares on the plantar pressure map, as shown in Figure 4a for three sensors and Figure 4b for six sensors. The CoP calculated from the moments and the total forces are shown by the small squares in Figure 4. The CoP moves under each foot from the heel to the forefoot.

We observed a good accordance between the Total Force estimate measured by the F-Scan and that calculated by the anatomical model using a set of three sensors, especially for normal and high-pressure levels (30 to 70 daN). For low-pressure levels, the relative errors are more important, probably due to the lack of calibration of the reference system.

**Figure 5:**
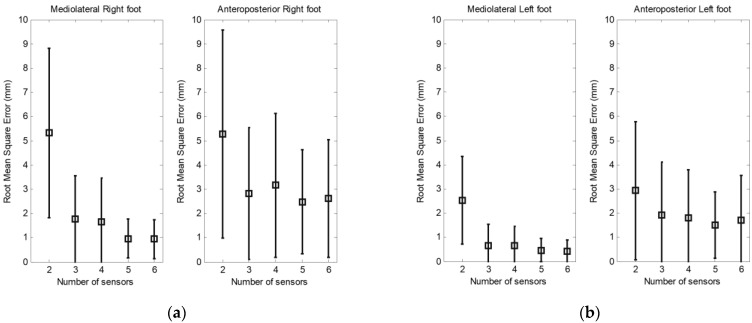
Root mean square error and standard deviation of mediolateral and anteroposterior CoP displacement underfoot (**a**) right and (**b**) left.

Figure 5 shows the root mean square (RMS) error and the standard deviation between the CoP measured by the F-Scan and that calculated by the anatomical model using a set of two to six sensors. The results are separated into the mediolateral and anteroposterior displacement of CoP under each foot. When we use two sensors, the error is about 5 mm under the right foot and 3 mm under the left foot.

**Figure 6:**
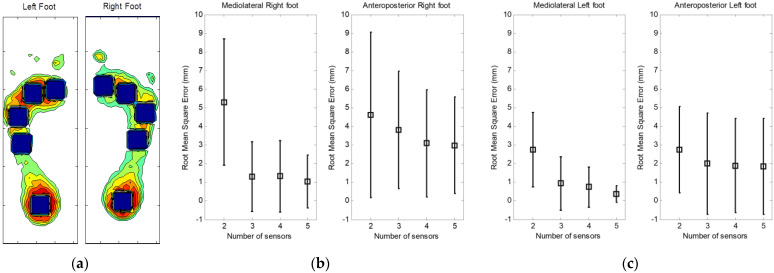
(**a**) Configuration of five large sensors (black squares) under each foot. (**b**) and (**c**) Root mean square error and standard deviation in mediolateral and anteroposterior CoP displacement under the right and left foot, respectively.

The same principle has been used in the case of large sensors. Figure 6a shows the position of five large underfoot sensors. One sensor is fixed under the heel, and four sensors completely cover the area under the forefoot and the arch. Figure 6b,c also shows the RMS and the standard deviation of mediolateral and anteroposterior CoP displacement under each foot for a set of two to five sensors.

### 3.2. Total Center of Pressure Measurement

**Figure 7:**
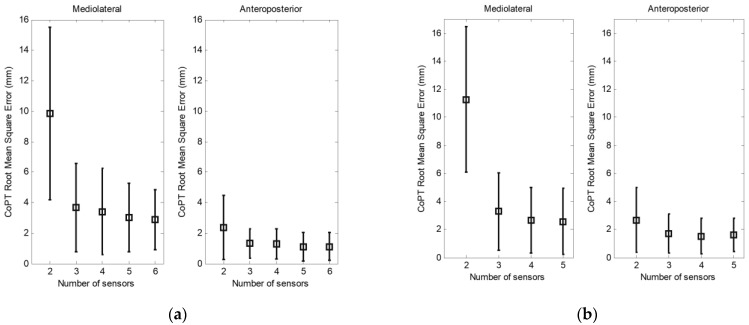
Effect of the number of (**a**) small sensors and (**b**) large sensors. Root mean square error and standard deviation in mediolateral and anteroposterior total CoP of the body.

In Figure 7, we present the RMS and the standard deviation between the CoPT measured by the sparse sensors and the F-Scan system when we use small (a) and large (b) sensors.

## 4. Discussion

### 4.1. Number of Sensors

Tests were conducted with varying numbers of sensors, from two to six sensors per foot. A minimum of two sensors per foot was assessed. We can verify that the results obtained with two sensors in the mediolateral direction show large errors when compared to those obtained with three or more sensors since the lateral component is not taken into account on each foot. If we first consider the effect of the number of sensors on the determination of the CoP under each foot, we can see that the calculated RMS is small when three sensors are used. We found only a slight improvement in the RMS errors when increasing the number of sensors from three to six.

Using the CoPT of the body as a criterion to evaluate the effect of the number of sensors, the results obtained confirm those observed when using the CoP under each foot. For example, in Figure 5, the highest error on the mediolateral CoPT is obtained when using two sensors. In contrast, with two sensors, the error is considerably smaller in the anteroposterior direction. This difference in the calculated error is due to the absence of an additional sensor to measure the mediolateral moments.

### 4.2. Position of Sensors

In the case of the barycentric CoP calculation, the position of the sensors under the foot remains an important criterion in the measurement of the plantar pressure. In addition, for more reliability, a high number of sensors are required to cover the total surface of the foot, which is the case of matrix insoles such as the F-Scan or the Pedar system.

In the case of our model, the sensors are placed in key locations under the foot, as described above. These include stationary positions such as heel and forefoot. However, as described in our method, the determination of the CoP is mathematically independent of the sensor position. Therefore, tests were carried out for different sensor positions under the foot. The results show a non-significant difference.

### 4.3. Surface of Sensors

Two sizes of sensors were investigated, small sensors with a surface of 1.5 × 1.5 cm^2^ and large sensors with a surface of 2.5 × 2.5 cm^2^. Comparing the two types of sensors, we observed a decrease in the mediolateral RMS errors with the small sensors. The RMS error is less than 4 mm in mediolateral displacement and approximately 1 mm in anteroposterior displacement when using three to six sensors.

### 4.4. Limitations

As described in [12], this model is able to describe total force and CoP displacements with good agreement. However, some limitations should be pointed out:

- The model considers only orthogonal pressure forces, regardless of transverse forces;
- Since the model is based on a healthy foot shape, adjustments are needed in case of complex biomechanical foot behavior;
- In addition, calibration (learning) is valid until the extracted foot shape parameters exhibit a significant drift. Then recalibration ought to be repeated.

## 5. Conclusions

We have presented a study on the robustness of our algorithm to the variation of the number, size, and position of sparse sensors applied to the measurement of the plantar pressure indicators. Based on a linear description of moments and total forces, the CoP is calculated from sparse pressure sensors. We have shown that insoles with a minimum of three pressure sensors per foot, combined with our anatomical foot model, provide a realistic estimation of CoP displacement under each foot and CoPT of the body. 

The results do not depend on the precise position of the sensors as long as the main pressure areas are equipped.

Small sensors provide better measurement results, contributing to power consumption reduction.

We emphasize that our study is based on an anatomical model of the foot, specifically adapted to each patient during a short learning period (2 to 5 s). 

Beyond the simplicity of this model, one of its main interests is that the parameters are directly meaningful by the physician, unlike other black box models such as PCA, SVM, or neural networks. 

Future work concerns the implementation of an ambulatory system with three sensors per insole. The amount of data to be collected would be reduced to only three pressure values per foot. The reduced amount of data would be transmitted to a podiatrist or locally processed within a smartphone processor.

## Figures and Tables

**Table 1:** Sensors positions.

# of Sensors	Heel	1st Metatarsal	2nd Metatarsal	3rd Metatarsal	5th Metatarsal	Arch
2	X			X		
3	X	X			X	
4	X	X		X	X	
5	X	X	X	X	X	
6	X	X	X	X	X	X

## Data Availability

Not applicable.
